# Structure and assembly of bacteriophage T4 head

**DOI:** 10.1186/1743-422X-7-356

**Published:** 2010-12-03

**Authors:** Venigalla B Rao, Lindsay W Black

**Affiliations:** 1 Department of Biology, The Catholic University of America, Washington, DC, USA; 2 Department of Biochemistry and Molecular Biology, University of Maryland Medical School, Baltimore, MD, USA

## Abstract

The bacteriophage T4 capsid is an elongated icosahedron, 120 nm long and 86 nm wide, and is built with three essential proteins; gp23*, which forms the hexagonal capsid lattice, gp24*, which forms pentamers at eleven of the twelve vertices, and gp20, which forms the unique dodecameric portal vertex through which DNA enters during packaging and exits during infection. The past twenty years of research has greatly elevated the understanding of phage T4 head assembly and DNA packaging. The atomic structure of gp24 has been determined. A structural model built for gp23 using its similarity to gp24 showed that the phage T4 major capsid protein has the same fold as that found in phage HK97 and several other icosahedral bacteriophages. Folding of gp23 requires the assistance of two chaperones, the *E. coli *chaperone GroEL and the phage coded gp23-specific chaperone, gp31. The capsid also contains two non-essential outer capsid proteins, Hoc and Soc, which decorate the capsid surface. The structure of Soc shows two capsid binding sites which, through binding to adjacent gp23 subunits, reinforce the capsid structure. Hoc and Soc have been extensively used in bipartite peptide display libraries and to display pathogen antigens including those from HIV, *Neisseria meningitides*, *Bacillus anthracis*, and FMDV. The structure of Ip1*, one of the components of the core, has been determined, which provided insights on how IPs protect T4 genome against the *E. coli *nucleases that degrade hydroxymethylated and glycosylated T4 DNA. Extensive mutagenesis combined with the atomic structures of the DNA packaging/terminase proteins gp16 and gp17 elucidated the ATPase and nuclease functional motifs involved in DNA translocation and headful DNA cutting. Cryo-EM structure of the T4 packaging machine showed a pentameric motor assembled with gp17 subunits on the portal vertex. Single molecule optical tweezers and fluorescence studies showed that the T4 motor packages DNA at a rate of up to 2000 bp/sec, the fastest reported to date of any packaging motor. FRET-FCS studies indicate that the DNA gets compressed during the translocation process. The current evidence suggests a mechanism in which electrostatic forces generated by ATP hydrolysis drive the DNA translocation by alternating the motor between tensed and relaxed states.

## Introduction

The T4-type bacteriophages are ubiquitously distributed in nature and occupy environmental niches ranging from mammalian gut to soil, sewage, and oceans. More than 130 such viruses that show similar morphological features as phage T4 have been described; from the T4 superfamily ~1400 major capsid protein sequences have been correlated to its 3D structure [1-3]. The features include large elongated (prolate) head, contractile tail, and a complex baseplate with six long, kinked tail fibers radially emanating from it. Phage T4 historically has served as an excellent model to elucidate the mechanisms of head assembly of not only T-even phages but of large icosahedral viruses in general, including the widely distributed eukaryotic viruses such as the herpes viruses. This review will focus on the advances in the past twenty years on the basic understanding of phage T4 head structure and assembly and the mechanism of DNA packaging. Application of some of this knowledge to develop phage T4 as a surface display and vaccine platform will also be discussed. The reader is referred to the comprehensive review by Black et al [4], for the early work on T4 head assembly.

### Structure of phage T4 capsid

**Figure 1:**
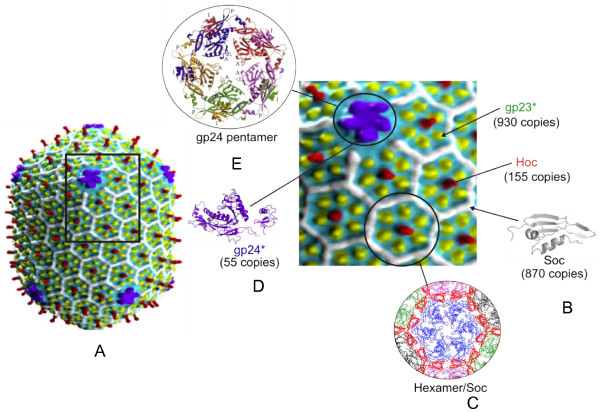
**Structure of the bacteriophage T4 head**. **A) **Cryo-EM reconstruction of phage T4 capsid [5]; the square block shows enlarged view showing gp23 (yellow subunits), gp24 (purple subunits), Hoc (red subunits) and Soc (white subunits); **B) **Structure of RB49 Soc; **C) **Structural model showing one gp23 hexamer (blue) surrounded by six Soc trimers (red). Neighboring gp23 hexamers are shown in green, black and magenta [28]; **D) **Structure of gp24 [6]; **E) **Structural model of gp24 pentameric vertex.

The overall architecture of the phage T4 head determined earlier by negative stain electron microscopy of the procapsid, capsid, and polyhead, including the positions of the dispensable Hoc and Soc proteins, has basically not changed as a result of cryo-electron microscopic structure determination of isometric capsids [5]. However, the dimensions of the phage T4 capsid and its inferred protein copy numbers have been slightly altered on the basis of the higher resolution cryo-electron microscopy structure. The width and length of the elongated prolate icosahedron [5] are T_end _= 13 laevo and T_mid _= 20 (86 nm wide and 120 nm long), and the copy numbers of gp23, Hoc and Soc are 960, 155, and 870, respectively (Figure 1).

The most significant advance was the crystal structure of the vertex protein, gp24, and by inference the structure of its close relative, the major capsid protein gp23 [6]. This ~0.3 nm resolution structure permits rationalization of head length mutations in the major capsid protein as well as of mutations allowing bypass of the vertex protein. The former map to the capsomer's periphery and the latter within the capsomer. It is likely that the special gp24 vertex protein of phage T4 is a relatively recent evolutionary addition as judged by the ease with which it can be bypassed. Cryo-electron microscopy showed that in the bypass mutants that substitute pentamers of the major capsid protein at the vertex, additional Soc decoration protein subunits surround these gp23* molecules, which does not occur in the gp23*-gp24* interfaces of the wild-type capsid [7]. Nevertheless, despite the rationalization of major capsid protein affecting head size mutations, it should be noted that these divert only a relatively small fraction of the capsids to altered and variable sizes. The primary determinant of the normally invariant prohead shape is thought to be its scaffolding core, which grows concurrently with the shell [4]. However, little progress has been made in establishing the basic mechanism of size determination or in determining the structure of the scaffolding core.

The gp24 and inferred gp23 structures are closely related to the structure of the major capsid protein of bacteriophage HK97, most probably also the same protein fold as the majority of tailed dsDNA bacteriophage major capsid proteins [8]. Interesting material bearing on the T-even head size determination mechanism is provided by "recent" T-even relatives of increased and apparently invariant capsid size, unlike the T4 capsid size mutations that do not precisely determine size (e.g. KVP40, 254 kb, apparently has a single T_mid _greater than the 170 kb T4 T_mid _= 20) [9]. However, few if any in depth studies have been carried out on these phages to determine whether the major capsid protein, the morphogenetic core, or other factors are responsible for the different and precisely determined volumes of their capsids.

### Folding of the major capsid protein gp23

Folding and assembly of the phage T4 major capsid protein gp23 into the prohead requires a special utilization of the GroEL chaperonin system and an essential phage co-chaperonin gp31. gp31 replaces the GroES co-chaperonin that is utilized for folding the 10-15% of *E. coli *proteins that require folding by the GroEL folding chamber. Although T4 gp31 and the closely related RB49 co-chaperonin CocO have been demonstrated to replace the GroES function for all essential *E. coli *protein folding, the GroES-gp31 relationship is not reciprocal; i.e. GroES cannot replace gp31 to fold gp23 because of special folding requirements of the latter protein [10,11]. The N-terminus of gp23 appears to strongly target associated fusion proteins to the GroEL chaperonin [12-14]. Binding of gp23 to the GroEL folding cage shows features that are distinct from those of most bound *E. coli *proteins. Unlike substrates such as RUBISCO, gp23 occupies both chambers of the GroEL folding cage, and only gp31 is able to promote efficient capped single "cis" chamber folding, apparently by creating a larger folding chamber [15]. On the basis of the gp24 inferred structure of gp23, and the structures of the GroES and gp31 complexed GroEL folding chambers, support for a critical increased chamber size to accommodate gp23 has been advanced as the explanation for the gp31 specificity [14]. However, since comparable size T-even phage gp31 homologs display preference for folding their own gp23s, more subtle features of the various T-even phage structured folding cages may also determine specificity.

### Structure of the packaged components of the phage T4 head

**Figure 2:**
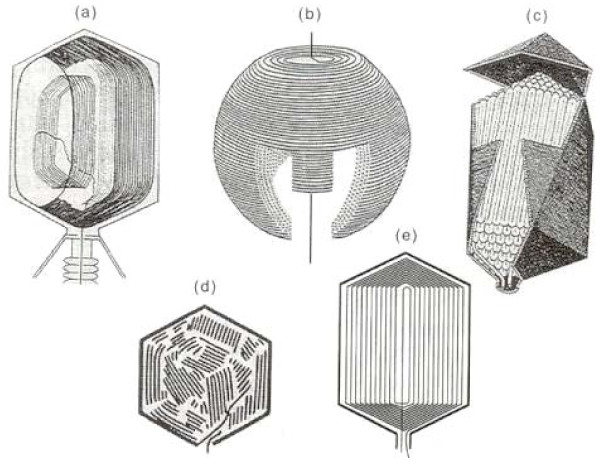
**Models of packaged DNA structure**. **a) **T4 DNA is packed longitudinally to the head-tail axis [91], unlike the transverse packaging in T7 capsids [16]**(b)**. Other models shown include spiral fold **(c)**, liquid-crystal **(d)**, and icosahedral-bend **(e)**. Both packaged T4 DNA ends are located in the portal [79]. For references and evidence bearing on packaged models see [19].

Packaged phage T4 DNA shares a number of general features with other tailed dsDNA phages: 2.5 nm side to side packing of predominantly B-form duplex DNA condensed to ~500 mg/ml. However, other features differ among phages; e.g. T4 DNA is packed in an orientation that is parallel to the head tail axis together with ~1000 molecules of imbedded and mobile internal proteins, unlike the DNA arrangement that traverses head-tail axis and is arranged around an internal protein core as seen in phage T7 [16]. Use of the capsid targeting sequence of the internal proteins allows encapsidation of foreign proteins such as GFP and staphylococcal nuclease within the DNA of active virus [17,18]. Digestion by the latter nuclease upon addition of calcium yields a pattern of short DNA fragments, predominantly a 160 bp repeat [19]. This pattern supports a discontinuous pattern of DNA packing such as in the icosahedral-bend or spiral-fold models. A number of proposed models (Figure 2) and experimental evidence bearing on these are summarized in [17].

**Figure 3:**
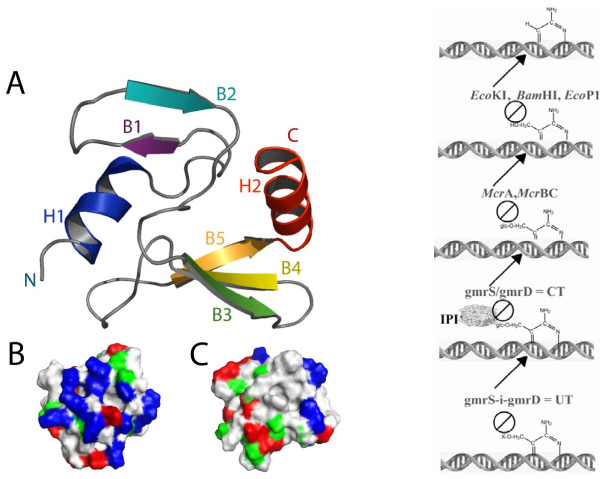
Structure and function of T4 internal protein I*. The NMR structure of IP1*, a highly specific inhibitor of the two-subunit CT (gmrS/gmrD) glucosyl-hmC DNA directed restriction endonuclease (right panel); shown are DNA modifications blocking such enzymes. The IPI* structure is compact with an asymmetric charge distribution on the faces (blue are basic residues) that may allow rapid DNA bound ejection through the portal and tail without unfolding-refolding.

In addition to the uncertain arrangement at the nucleotide level of packaged phage DNA, the structure of other internal components is poorly understood in comparison to surface capsid proteins. The internal protein I* (IPI*) of phage T4 is injected to protect the DNA from a two subunit gmrS + gmrD glucose modified restriction endonuclease of a pathogenic *E. coli *that digests glucosylated hydroxymethylcytosine DNA of T-even phages [20,21]. The 76-residue proteolyzed mature form of the protein has a novel compact protein fold consisting of two beta sheets flanked with N- and C-terminal alpha helices, a structure that is required for its inhibitor activity that is apparently due to binding the gmrS/gmrD proteins (Figure 3) [22]. A single chain gmrS/gmrD homolog enzyme with 90% identity in its sequence to the two subunit enzyme has evolved IPI* inhibitor immunity. It thus appears that the phage T-evens have co-evolved with their hosts, a diverse and highly specific set of internal proteins to counter the hmC modification dependent restriction endonucleases. Consequently the internal protein components of the T-even phages are a highly diverse set of defense proteins against diverse attack enzymes with only a conserved capsid targeting sequence (CTS) to encapsidate the proteins into the precursor scaffolding core [23].

Genes 2 and 4 of phage T4 likely are associated in function and gp2 was previously shown by Goldberg and co-workers to be able to protect the ends of mature T4 DNA from the recBCD exonuclease V, likely by binding to the DNA termini. The gp2 protein has not been identified within the phage head because of its low abundance but evidence for its presence in the head comes from the fact that gp2 can be added to gp2 deficient full heads to confer exonuclease V protection. Thus gp2 affects head-tail joining as well as protecting the DNA ends likely with as few as two copies per particle binding the two DNA ends [24].

Solid state NMR analysis of the phage T4 particle shows the DNA is largely B form and allows its electrostatic interactions to be tabulated [25]. This study reveals high resolution interactions bearing on the internal structure of the phage T4 head. The DNA phosphate negative charge is balanced among lysyl amines, polyamines, and mono and divalent cations. Interestingly, among positively charged amino acids, only lysine residues of the internal proteins were seen to be in contact with the DNA phosphates, arguing for specific internal protein DNA structures. Electrostatic contributions from internal proteins and polyamines' interactions with DNA entering the prohead to the packaging motor were proposed to account for the higher packaging rates achieved by the phage T4 packaging machine when compared to that of Phi29 and lambda phages.

### Display on capsid

In addition to the essential capsid proteins, gp23, gp24, and gp20, the T4 capsid is decorated with two non-essential outer capsid proteins: Hoc (highly antigenic outer capsid protein), a dumbbell shaped monomer at the center of each gp23 hexon, up to 155 copies per capsid (39 kDa; red subunits); and Soc (small outer capsid protein), a rod-shaped molecule that binds between gp23 hexons, up to 870 copies per capsid (9 kDa; white subunits) (Figure 1). Both Hoc and Soc are dispensable, and bind to the capsid after the completion of capsid assembly [26,27]. Null (amber or deletion) mutations in either or both the genes do not affect phage production, viability, or infectivity.

The structure of Soc has recently been determined [28]. It is a tadpole shaped molecule with two binding sites for gp23*. Interaction of Soc to the two gp23 molecules glues adjacent hexons. Trimerization of the bound Soc molecules results in clamping of three hexons, and 270 such clamps form a cage reinforcing the capsid structure. Soc assembly thus provides great stability to phage T4 to survive under hostile environments such as extreme pH (pH 11), high temperature (60°C), osmotic shock, and a host of denaturing agents. Soc-minus phage lose viability at pH10.6 and addition of Soc enhances its survival by ~10^4^-fold. On the other hand, Hoc does not provide significant additional stability. With its Ig-like domains exposed on the outer surface, Hoc may interact with certain components of the bacterial surface, providing additional survival advantage (Sathaliyawala and Rao, unpublished results).

The above properties of Hoc and Soc are uniquely suited to engineer the T4 capsid surface by arraying pathogen antigens. Ren et al and Jiang et al developed recombinant vectors that allowed fusion of pathogen antigens to the N- or C-termini of Hoc and Soc [29-32]. The fusion proteins were expressed in *E. coli *and upon infection with *hoc*^-^*soc*^- ^phage, the fusion proteins assembled on the capsid. The phages purified from the infected extracts are decorated with the pathogen antigens. Alternatively, the fused gene can be transferred into T4 genome by recombinational marker rescue and infection with the recombinant phage expresses and assembles the fusion protein on the capsid as part of the infection process. Short peptides or protein domains from a variety of pathogens, *Neisseria meningitides *[32], polio virus [29], HIV [29,33], swine fever virus [34], and foot and mouth disease virus [35], have been displayed on T4 capsid using this approach.

The T4 system can be adapted to prepare bipartite libraries of randomized short peptides displayed on T4 capsid Hoc and Soc and use these libraries to "fish out" peptides that interact with the protein of interest [36]. Biopanning of libraries by the T4 large packaging protein gp17 selected peptides that matches with the sequences of proteins that are thought to interact with p17. Of particular interest was the selection of a peptide that matched with the T4 late sigma factor, gp55. The gp55 deficient extracts packaged concatemeric DNA about 100-fold less efficiently suggesting that the gp17 interaction with gp55 helps loading the packaging terminase onto the viral genome [36,37].

**Figure 4:**
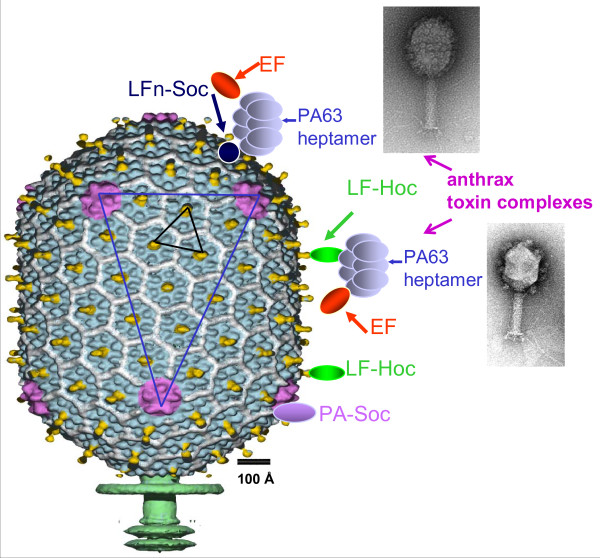
**In vitro display of antigens on bacteriophage T4 capsid**. Schematic representation of the T4 capsid decorated with large antigens, PA (83 kDa) and LF (89 kDa), or hetero-oligomeric anthrax toxin complexes through either Hoc or Soc binding [39,41]. See text for details. The insets show electron micrographs of T4 phage with the anthrax toxin complexes displayed through Soc (top) or Hoc (bottom). Note the copy number of the complexes is lower with the Hoc display than with the Soc display.

An in vitro display system has been developed taking advantage of the high affinity interactions between Hoc or Soc and the capsid (Figure 4) [38,39]. In this system, the pathogen antigen fused to Hoc or Soc with a hexa-histidine tag was overexpressed in *E. coli *and purified. The purified protein was assembled on *hoc*^-^*soc*^- ^phage by simply mixing the purified components. This system has certain advantages over the in vivo display: i) a functionally well characterized and conformationally homogeneous antigen is displayed on the capsid; ii) the copy number of displayed antigen can be controlled by altering the ratio of antigen to capsid binding sites; and iii) multiple antigens can be displayed on the same capsid. This system was used to display full-length antigens from HIV [33] and anthrax [38,39] that are as large as 90 kDa.

All 155 Hoc binding sites can be filled with anthrax toxin antigens, protective antigen (PA, 83 kDa), lethal factor (LF, 89 kDa), or edema factor (EF, 90 kDa) [36,40]. Fusion to the N-terminus of Hoc did not affect the apparent binding constant (*K*_*d*_) or the copy number per capsid (*B*_*max*_), but fusion to the C-terminus reduced the *K*_*d *_by 500-fold [32,40]. All 870 copies of Soc binding sites can be filled with Soc-fused antigens but the size of the fused antigen must be ~30 kDa or less; otherwise, the copy number is significantly reduced [39]. For example, the 20-kDa PA domain-4 and the 30 kDa LFn domain fused to Soc can be displayed to full capacity. An insoluble Soc-HIV gp120 V3 loop domain fusion protein with a 43 aa C-terminal addition could be refolded and bound with ~100% occupancy to mature phage head type-polyheads [29]. Large 90 kDa anthrax toxins can also be displayed but the *B*_*max *_is reduced to about 300 presumably due to steric constraints. Antigens can be fused to either the N- or C-terminus, or both the termini of Soc simultaneously, without significantly affecting the *K*_*d *_or *B*_*max*_. Thus, as many as 1895 antigen molecules or domains can be attached to each capsid using both Hoc and Soc [39].

The in vitro system offers novel avenues to display macromolecular complexes through specific interactions with the already attached antigens [41]. Sequential assembly was performed by first attaching LF-Hoc and/or LFn-Soc to *hoc*^-^*soc*^- ^phage and exposing the N-domain of LF on the surface. Heptamers of PA were then assembled through interactions between the LFn domain and the N-domain of cleaved PA (domain 1' of PA63). EF was then attached to the PA63 heptamers, completing the assembly of the ~700 kDa anthrax toxin complex on phage T4 capsid (Figure 4). CryoEM reconstruction shows that native PA63_(7)_-LFn_(3) _complexes are assembled in which three adjacent capsid-bound LFn "legs" support the PA63 heptamers [42]. Additional layers of proteins can be built on the capsid through interactions with the respective partners.

One of the main applications of the T4-antigen particles is their potential use in vaccine delivery. A number of independent studies showed that the T4-displayed particulate antigens without any added adjuvant elicit strong antibody responses, and to a lesser extent cellular responses [28,32]. The 43 aa V3 loop of HIV gp120 fused to Soc displayed on T4 phage was highly immunogenic in mice and induced anti-gp120 antibodies; so was the Soc-displayed IgG anti-EWL [29]. The Hoc fused 183 aa N-terminal portion of HIV CD4 receptor protein is displayed in active form. Strong anthrax lethal-toxin neutralization titers were elicited upon immunization of mice and rabbits with phage T4-displayed PA either through Hoc or Soc ([38,40], Rao, unpublished data). When multiple anthrax antigens were displayed, immune responses against all the displayed antigens were elicited [40]. The T4 particles displaying PA and LF, or those displaying the major antigenic determinant cluster mE2 (123 aa) and the primary antigen E2 (371 aa) of the classical swine fever virus elicited strong antibody titers [34]. Furthermore, mice immunized with the Soc displayed foot and mouth disease virus (FMDV) capsid precursor polyprotein (P1, 755 aa) and proteinase 3C (213 aa) were completely protected upon challenge with a lethal dose of FMDV [34,35]. Pigs immunized with a mixture of T4-P1 and T4-3C particles were also protected when these animals were co-housed with FMDV infected pigs. In another type of application, T4-displayed mouse Flt4 tumor antigen elicited anti-Flt4 antibodies and broke immune tolerance to self-antigens. These antibodies provided antitumor and anti-metastasis immunity in mice [43].

The above studies provide abundant evidence that the phage T4 nanoparticle platform has the potential to engineer human as well as veterinary vaccines.

### DNA packaging

Two nonstructural terminase proteins, gp16 (18 kDa) and gp17 (70 kDa), link head assembly and genome processing [44-46]. These proteins are thought to form a hetero-oligomeric complex, which recognizes the concatemeric DNA and makes an endonucleolytic cut (hence the name "terminase"). The terminase-DNA complex docks on the prohead through gp17 interactions with the special portal vertex formed by the dodecameric gp20, thus assembling a DNA packaging machine. The gp49 EndoVII Holliday structure resolvase also specifically associates with the portal dodecamer thereby positioning this enzyme to repair packaging-arrested branched-structure-containing concatemers [47]. The ATP-fueled machine translocates DNA into the capsid until the head is full, equivalent to about 1.02 times the genome length (171 kb). The terminase dissociates from the packaged head, makes a second cut to terminate DNA packaging and attaches the concatemeric DNA to another empty head to continue translocation in a processive fashion. Structural and functional analyses of the key parts of the machine - gp16, gp17, and gp20 - as described below, led to models for the packaging mechanism.

#### gp16

gp16, the 18 kDa small terminase subunit, is dispensable for packaging linear DNA in vitro but it is essential in vivo; amber mutations in gene 16 accumulate empty proheads resulting in null phenotype [37,48].

**Figure 5:**
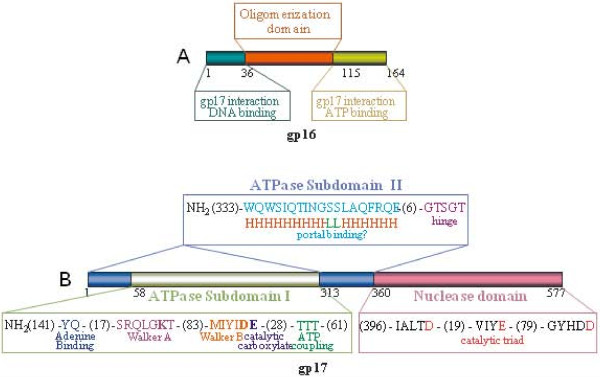
**Domains and motifs in phage T4 terminase proteins**. Schematic representation of domains and motifs in the small terminase protein gp16. **A) **and the large terminase protein gp17 **(B)**. The functionally critical amino acids are shown in bold. Numbers represent the number of amino acids in the respective coding sequence. For further detailed explanations of the functional motifs, refer to [46] and [51].

Mutational and biochemical analyses suggest that gp16 is involved in the recognition of viral DNA [49,50] and regulation of gp17 functions [51]. gp16 is predicted to contain three domains, a central domain that is important for oligomerization, and N- and C-terminal domains that are important for DNA binding, ATP binding, and/or gp17-ATPase stimulation [51,52] (Figure 5). gp16 forms oligomeric single and side-by-side double rings, each ring having a diameter of ~8 nm with ~2 nm central channel [49,52]. Recent mass spectrometry determination shows that the single and double rings are 11-mers and 22-mers respectively [53]. A number of *pac *site phages produce comparable small terminase subunit multimeric ring structures. Sequence analyses predict 2-3 coiled coil motifs in gp16 [48]. All the T4 family gp16s as well as other phage small terminases consist of one or more coiled coil motifs, consistent with their propensity to form stable oligomers. Oligomerization presumably occurs through parallel coiled-coil interactions between neighboring subunits. Mutations in the long central α-helix of T4 gp16 that perturb coiled coil interactions lose the ability to oligomerize [48].

gp16 appears to oligomerize following interaction with viral DNA concatemer, forming a platform for the assembly of the large terminase gp17. A predicted helix-turn-helix in the N-terminal domain is thought to be involved in DNA-binding [49,52]. The corresponding motif in the phage lambda small terminase protein, gpNu1, has been well characterized and demonstrated to bind the DNA. In vivo genetic studies and in vitro DNA binding studies show that a 200 bp 3'-end sequence of gene 16 is a preferred "*pac*" site for gp16 interaction [49,50]. It was proposed that the stable gp16 double rings were two turn lock washers that constituted the structural basis for synapsis of two *pac *site DNAs. This could promote the gp16 dependent gene amplifications observed around the *pac *site that can be selected in *alt*- mutants that package more DNA; such synapsis could function as a gauge of DNA concatemer maturation [54-56].

gp16 stimulates the gp17-ATPase activity by > 50-fold [57,58]. Stimulation is likely via oligomerization of gp17 which does not require gp16 association [58]. gp16 also stimulates in vitro DNA packaging activity in the crude system where phage infected extracts containing all the DNA replication/transcription/recombination proteins are present [57,59], but inhibits the packaging activity in the defined system where only two purified components, proheads and gp17, are present [37,60]. It stimulates gp17-nuclease activity when T4 transcription factors are also present but inhibits the nuclease in a pure system [51]. gp16 also inhibits gp17's binding to DNA [61]. Both the N- and C-domains are required for ATPase stimulation or nuclease inhibition [51]. Maximum effects were observed at a ratio of approximately 8 gp16 molecules to 1 gp17 molecule suggesting that in the holoterminase complex one gp16 oligomer interacts with one gp17 monomer [62].

gp16 contains an ATP binding site with broad nucleotide specificity [49,51], however it lacks the canonical nucleotide binding signatures such as Walker A and Walker B [52]. No correlation was evident between nucleotide binding and gp17-ATPase stimulation or gp17-nuclease inhibition. Thus it is unclear what the role of ATP binding plays in gp16 function.

The evidence thus far suggests that gp16 is a regulator of the DNA packaging machine, modulating the ATPase, translocase, and nuclease activities of gp17. Although the regulatory functions can be dispensable for in vitro DNA packaging, these are essential in vivo to coordinate the packaging process and produce an infectious virus particle [51].

#### gp17

gp17 is the 70 kDa large subunit of the terminase holoenzyme and the motor protein of the DNA packaging machine. gp17 consists of two functional domains (Figure 5); an N-terminal ATPase domain having the classic ATPase signatures such as Walker A, Walker B, and catalytic carboxylate, and a C-terminal nuclease domain having a catalytic metal cluster with conserved aspartic and glutamic acid residues coordinating with Mg [62].

gp17 alone is sufficient to package DNA in vitro. gp17 exhibits a weak ATPase activity (K_cat _= ~1-2 ATPs hydrolyzed per gp17 molecule/min), which is stimulated by > 50-fold by the small terminase protein gp16 [57,58]. Any mutation in the predicted catalytic residues of the N-terminal ATPase center results in a loss of stimulated ATPase and DNA packaging activities [63]. Even subtle conservative substitutions such as aspartic acid to glutamic acid and *vice versa *in the Walker B motif resulted in complete loss of DNA packaging suggesting that this ATPase provides energy for DNA translocation [64,65].

The ATPase domain also exhibits DNA binding activity, which may be involved in the DNA cutting and translocation functions of the packaging motor. There is genetic evidence that gp17 may interact with gp32 [66,67], but highly purified preparations of gp17 do not show appreciable affinity for ss or ds DNA. There seem to be complex interactions between the terminase proteins, the concatemeric DNA, and the DNA replication/recombination/repair and transcription proteins that transition the DNA metabolism into the packaging phase [37].

**Figure 6:**
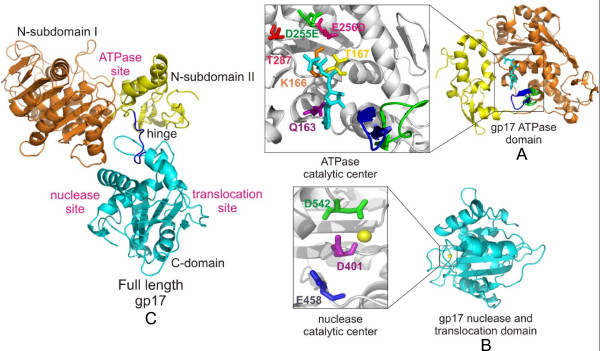
**Structures of the T4 packaging motor protein, gp17**. Structures of the ATPase domain: **A) **nuclease/translocation domain; **B)**, and full-length gp17; **C)**. Various functional sites and critical catalytic residues are labeled. See references [68] and [74] for further details.

One of the ATPase mutants, the DE-ED mutant in which the sequence of Walker B and catalytic carboxylate was reversed, showed tighter binding to ATP than the wild-type gp17 but failed to hydrolyze ATP [64]. Unlike the wild-type gp17 or the ATPase domain which failed to crystallize, the ATPase domain with the ED mutation crystallized readily, probably because it trapped the ATPase in an ATP-bound conformation. The X-ray structure of the ATPase domain was determined up to 1.8 Å resolution in different bound states; apo, ATP-bound, and ADP-bound [68]. It is a flat structure consisting of two subdomains; a large subdomain I (NsubI) and a smaller subdomain II (NsubII) forming a cleft in which ATP binds (Figure 6A). The NsubI consists of the classic nucleotide binding fold (Rossmann fold), a parallel β-sheet of six β-strands interspersed with helices. The structure showed that the predicted catalytic residues are oriented into the ATP pocket, forming a network of interactions with bound ATP. These also include an arginine finger that is proposed to trigger βγ-phosphoanhydride bond cleavage. In addition, the structure showed the movement of a loop near the adenine binding motif in response to ATP hydrolysis, which may be important for transduction of ATP energy into mechanical motion.

gp17 exhibits a sequence nonspecific endonuclease activity [69,70]. Random mutagenesis of gene 17 and selection of mutants that lost nuclease activity identified a histidine-rich site in the C-terminal domain being critical for DNA cleavage [71]. Extensive site-directed mutagenesis of this region combined with the sequence alignments identified a cluster of conserved aspartic acid and glutamic acid residues that are essential for DNA cleavage [72]. Unlike the ATPase mutants, these mutants retained the gp16-stimulated ATPase activity as well as the DNA packaging activity as long as the substrate is a linear molecule. However these mutants fail to package circular DNA as they are defective in cutting DNA that is required for packaging initiation.

The structure of the C-terminal nuclease domain from a T4-family phage, RB49, which has 72% sequence identity to the T4 C-domain, was determined to 1.16Å resolution [73] (Figure 6B). It has a globular structure consisting mostly of anti-parallel β-strands forming an RNase H fold that is found in resolvases, RNase Hs and integrases. As predicted from the mutagenesis studies, the structures showed that the residues D401, E458 and D542 form a catalytic triad coordinating with Mg ion. In addition the structure showed the presence of a DNA binding groove lined with a number of basic residues. The acidic catalytic metal center is buried at one end of this groove. Together, these form the nuclease cleavage site of gp17.

The crystal structure of the full-length T4 gp17 (ED mutant) was determined to 2.8Å resolution (Figure 6C) [74]. The N- and C-domain structures of the full-length gp17 superimpose with those solved using individually crystallized domains with only minor deviations. The full-length structure however has additional features that are relevant to the mechanism. A flexible "hinge" or "linker" connects the ATPase and nuclease domains. Previous biochemical studies showed that splitting gp17 into two domains at the linker retained the respective ATPase and nuclease functions but DNA translocation activity was completely lost [62]. Second, the N- and C-domains have a > 1000 square Å complementary surface area consisting of an array of five charged pairs and hydrophobic patches [74]. Third, the gp17 has a bound phosphate ion in the crystal structure. Docking of B-form DNA guided by shape and charge complementarity with one of the DNA phosphates superimposed on the bound phosphate aligns a number of basic residues, lining what appears to be a shallow translocation groove. Thus the C-domain appears to have two DNA grooves on different faces of the structure, one that aligns with the nuclease catalytic site and the second that aligns with the translocating DNA (Figure 6). Mutation of one of the groove residues (R406) showed a novel phenotype; loss of DNA translocation activity but the ATPase and nuclease activities are retained.

#### Motor

A functional DNA packaging machine could be assembled by mixing proheads and purified gp17. gp17 assembles into a packaging motor through specific interactions with the portal vertex [75] and such complexes can package the 171 kb phage T4 DNA, or any linear DNA [37,60]. If short DNA molecules are added as the DNA substrate, the motor keeps packaging DNA until the head is full [76].

Packaging can be studied in real time either by fluorescence correlation spectroscopy [77] or by optical tweezers [78]. The translocation kinetics of rhodamine (R6G) labeled 100 bp DNA was measured by determining the decrease in diffusion coefficient as the DNA gets confined inside the capsid. Fluorescence resonance energy transfer between the green fluorescent protein labeled proteins within the prohead interior and the translocated rhodamine-labeled DNA confirmed the ATP-powered movement of DNA into the capsid and the packaging of multiple segments per procapsid [77]. Analysis of FRET dye pair end labeled DNA substrates showed that upon packaging the two ends of the packaged DNA were held 8-9 nm apart in the procapsid, likely fixed in the portal channel and crown, and suggesting that a loop rather than an end of DNA is translocated following initiation at an end [79].

In the optical tweezers system, the prohead-gp17 complexes were tethered to a microsphere coated with capsid protein antibody, and the biotinylated DNA is tethered to another microsphere coated with streptavidine. The microspheres are brought together into near contact, allowing the motor to capture the DNA. Single packaging events were monitored and the dynamics of the T4 packaging process were quantified [78]. The T4 motor, like the Phi29 DNA packaging motor, generates forces as high as ~60 pN, which is ~20-25 times that of myosin ATPase and a rate as high as ~2000 bp/sec, the highest recorded to date. Slips and pauses occur but these are relatively short and rare and the motor recovers and recaptures DNA continuing translocation. The high rate of translocation is in keeping with the need to package the 171 kb size T4 genome in about 5 minutes. The T4 motor generates enormous power; when an external load of 40 pN was applied, the T4 motor translocates at a speed of ~380 bp/sec. When scaled up to a macromotor, the T4 motor is approximately twice as powerful as a typical automobile engine.

**Figure 7:**
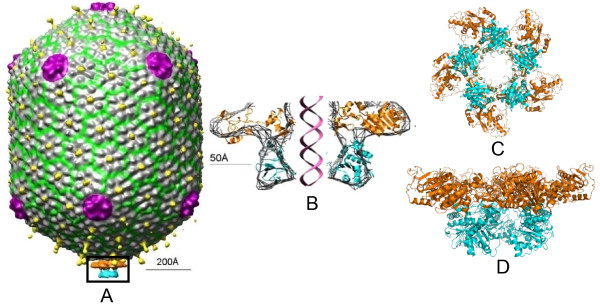
**Structure of the T4 DNA packaging machine**. **A) **Cryo-EM reconstruction of the phage T4 DNA packaging machine showing the pentameric motor assembled at the special portal vertex. **B-D) **Cross section, top and side views of the pentameric motor respectively, by fitting the X-ray structures of the gp17 ATPase and nuclease/translocation domains into the cryo-EM density.

CryoEM reconstruction of the packaging machine showed two rings of density at the portal vertex [74] (Figure 7). The upper ring is flat, resembling the ATPase domain structure and the lower ring is spherical, resembling the C-domain structure. This was confirmed by docking of the X-ray structures of the domains into the cryoEM density. The motor has pentamer stoichiometry, with the ATP binding surface facing the portal and interacting with it. It has an open central channel that is in line with the portal channel and the translocation groove of the C-domain faces the channel. There are minimal contacts between the adjacent subunits suggesting that the ATPases may fire relatively independently during translocation.

Unlike the cryoEM structure where the two lobes (domains) of the motor are separated ("relaxed" state), the domains in the full-length gp17 are in close contact ("tensed" state) [74]. In the tensed state, the subdomain II of ATPase is rotated by 6° degrees and the C-domain is pulled upwards by 7Å, equivalent to 2 bp. The "arginine finger" located between subI and NsubII is positioned towards the βγ phosphates of ATP and the ion pairs are aligned.

#### Mechanism

Of many models proposed to explain the mechanism of viral DNA translocation, the portal rotation model attracted the most attention. According to the original and subsequent rotation models, the portal and DNA are locked like a nut and bolt [80,81]. The symmetry mismatch between the 5-fold capsid and 12-fold portal means that only one portal subunit aligns with one capsid subunit at any given time, causing the associated terminase-ATPase to fire causing the portal, the nut, to rotate, allowing the DNA, the bolt, to move into the capsid. Indeed, the overall structure of the dodecameric portal is well conserved in numerous bacteriophages and even in HSV, despite no significant sequence similarity. However, the X-ray structures of Phi29 and SPP1 portals did not show any rigid groove-like features that are complementary to the DNA structure [81-83]. The structures are nevertheless consistent with the proposed portal rotation and newer, more specific, models such as the rotation-compression-relaxation [81], electrostatic gripping [82], and molecular lever [83], have been proposed.

Protein fusions to either the N or C terminal end of the portal protein could be incorporated into up to ~one-half of the dodecamer positions without loss of prohead function. As compared to wild-type, portals containing C-terminal GFP fusions lock the proheads into the unexpanded conformation unless terminase packages DNA, suggesting that the portal plays a central role in controlling prohead expansion. Expansion is required to protect the packaged DNA from nuclease but not for packaging itself as measured by FCS [84]. Moreover retention of DNA packaging function of such portals argues against the portal rotation model, since rotation would require that the bulky C-terminal GFP fusion proteins within the capsid rotate through the densely packaged DNA. A more direct test tethered the portal to the capsid through Hoc interactions [85]. Hoc is a nonessential T4 outer capsid protein that binds as a monomer at the center of the major capsid protein hexon (see above; Figure 1). Hoc binding sites are not present in the unexpanded proheads but are exposed following capsid expansion. To tether the portal, unexpanded proheads were first prepared with 1 to 6 of the 12 portal subunits replaced by the N-terminal Hoc-portal fusion proteins. The proheads were then expanded in vitro to expose Hoc binding sites. The Hoc portion of the portal fusion would bind to the center of the nearest hexon, tethering 1 to 5 portal subunits to the capsid. The Hoc-capsid interaction is thought to be irreversible and thus should prevent the rotation of the portal. If portal rotation were to be central to DNA packaging, the tethered expanded proheads should show very little or no packaging activity. However, the efficiency and rate of packaging of tethered proheads were comparable to those of wild-type proheads, suggesting that portal rotation is not an obligatory requirement for packaging [85]. This was more recently confirmed by single molecule fluorescence spectroscopy of actively packaging Phi29 packaging complexes [86].

In the second class of models, the terminase not only provides the energy but also actively translocates DNA [87]. Conformational changes in the terminase domains cause changes in the DNA binding affinity resulting in binding and releasing DNA, reminiscent of the inchworm-type translocation by helicases. gp17 and numerous large terminases possess an ATPase coupling motif that is commonly present in helicases and translocases [87]. Mutations in the coupling motif present at the junction of NSubI and NSubII result in loss of ATPase and DNA packaging activities.

**Figure 8:**
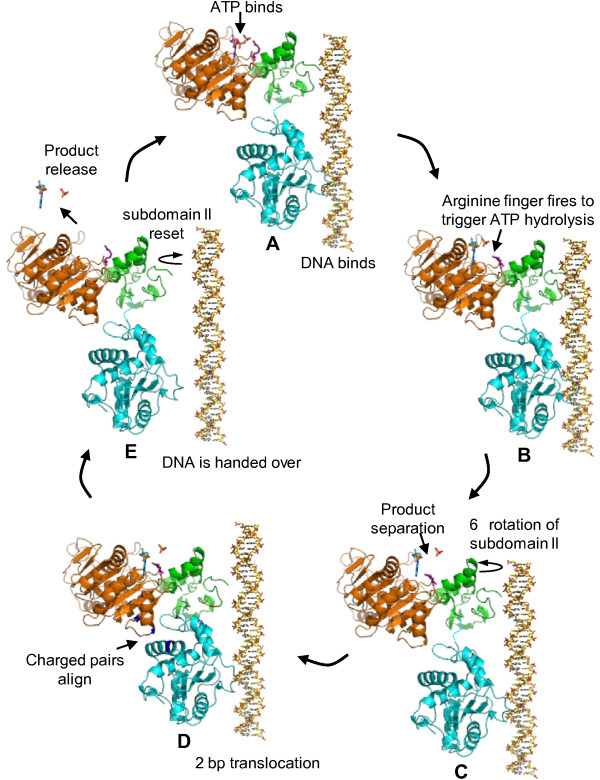
**A model for the electrostatic force driven DNA packaging mechanism**. Schematic representation showing the sequence of events that occur in a single gp17 molecule to translocate 2 bp of DNA (see the text and reference [74] for details).

The cryoEM and X-ray structures (Figure 7) combined with the mutational analyses led to the postulation of a terminase-driven packaging mechanism [74]. The pentameric T4 packaging motor can be considered to be analogous to a five cylinder engine. It consists of an ATPase center in NsubI, which is the engine that provides energy. The C-domain has a translocation groove, which is the wheel that moves DNA. The smaller NsubII is the transmission domain, coupling the engine to the wheel via a flexible hinge. The arginine finger is a spark plug that fires ATPase when the motor is locked in the firing mode. Charged pairs generate electrostatic force by alternating between relaxed and tensed states (Figure 8). The nuclease groove faces away from translocating DNA and is activated when packaging is completed.

In the relaxed conformational state (cryoEM structure), the hinge is extended (Figure 8). Binding of DNA to the translocation groove and of ATP to NsubI locks the motor in translocation mode (A) and brings the arginine finger into position, firing ATP hydrolysis (B). The repulsion between the negatively charged ADP(3-) and Pi(3-) drive them apart, causing NsubII to rotate by 6° (C), aligning the charge pairs between the N- and C-domains. This generates electrostatic force, attracting the C-domain-DNA complex and causing 7Å upward movement, the tensed conformational state (X-ray structure) (D). Thus 2 bp of DNA is translocated into the capsid in one cycle. Product release and loss of 6 negative charges causes NsubII to rotate back to original position, misaligning the ion pairs and returning the C-domain to the relaxed state (E).

Translocation of 2 bp would bring the translocation groove of the adjacent subunit into alignment with the backbone phosphates. DNA is then handed over to the next subunit, by the matching motor and DNA symmetries. Thus, ATPase catalysis causes conformational changes which generate electrostatic force, which is then converted to mechanical force. The pentameric motor translocates 10 bp (one turn of the helix) when all five gp17 subunits fire in succession, bringing the first gp17 subunit once again in alignment with the DNA phosphates. Synchronized orchestration of the motor's movements translocates DNA up to ~2000 bp/sec.

**Figure 9:**
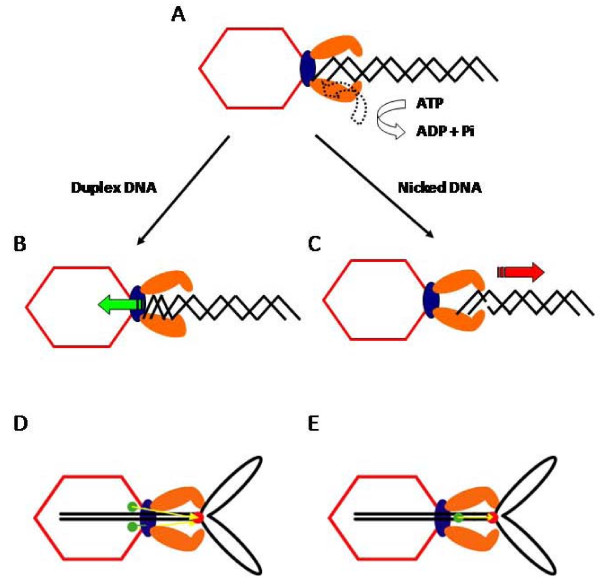
**A model for the torsional compression portal-DNA-grip-and-release packaging mechanism**. **A-C) **Short nicked or other abnormal structure containing DNA substrates are released from the motor. **D) **Leader containing Y-DNA substrates are retained by the motor and are anchored in the procapsid in proximity to portal GFP fusions; and **E) **compression of the Y-stem B segment in the stalled complex is observed by FRET [88,89]

Short (< 200 bp) DNA substrate translocation by gp17 is blocked by nicks, gaps, hairpin ends, RNA-containing duplexes, 20-base mismatches and D-loops, but not by 10-base internal mismatches [88]. Packaging of DNAs as short as 20 bp and initiation at almost any type DNA end suggests translocation rather than initiation deficiency of these short centrally nicked or gapped DNAs. Release from the motor of 100 bp nicked DNA segments supported a torsional compression portal-DNA-grip-and-release mechanism, where the portal grips the DNA while the gp17 imparts a linear force that may be stored in the DNA as compression or dissipated by a nick (Figure 9). Use of a DNA leader joined to a Y-DNA structure showed packaging of the leader segment; the Y-junction was arrested in proximity to a prohead portal containing GFP fusions, allowing FRET transfer between the Y-junction located dye molecule and the portal GFPs [89] (Figure 9D). Comparable stalled Y-DNA substrates containing FRET-pair dyes in the Y-stem showed that the motor compresses the stem held in the portal channel by 22-24% (Figure 9E. This finding supports the proposal that torsional compression of B DNA by the terminase motor by a portal-DNA-grip-and-release mechanism helps to drive translocation [88]. Attaching a longer DNA leader to the Y-DNA allows such abnormal structure substrates to be anchored in the procapsid for successful translocation, most likely by multiple motor cycles [89]. Differences in DNA substrate size may at least in part account for much less stringent DNA structural requirements measured in the Phi29 packaging system [90].

## Conclusions

It is clear from the above discussion that major advances have been made in recent years on the understanding of the phage T4 capsid structure and mechanism of DNA packaging. These advances, by combining genetics and biochemistry with structure and biophysics, set the stage to probe the packaging mechanism with even greater depth and precision. It is reasonable to hope that this would lead to the elucidation of catalytic cycle, mechanistic details, and motor dynamics to near atomic resolution. The accumulated and emerging basic knowledge should also lead to medical applications such as the development of vaccines and phage therapy.

## List of abbreviations

EF: edema factor; EM: electron microscopy; FCS: fluorescence correlation spectroscopy; FMDV: foot and mouth disease virus; FRET: fluorescence resonance energy transfer; gp: gene product; HIV: human immunodeficiency virus; Hoc: highly antigenic outer capsid protein; IP: internal protein; LF: lethal factor; PA: protective antigen; Soc: small outer capsid protein;

## Competing interests

The authors declare that they have no competing interests.

## Authors' contributions

VR and LWB made equal contributions to drafts of this review. Both authors revised all sections of the article and read and approved the final manuscript.
